# When Frail Older People Relocate in Very Old Age, Who Makes the Decision?

**DOI:** 10.1093/geroni/igz030

**Published:** 2019-09-06

**Authors:** Fiona Scheibl, Morag Farquhar, Jackie Buck, Stephen Barclay, Carol Brayne, Jane Fleming

**Affiliations:** 1 Cambridge Institute of Public Health, University of Cambridge, UK, Norwich, UK; 2 School of Health Sciences, University of East Anglia, Norwich, UK

**Keywords:** Dementia, Relocation, Oldest old, Decision making

## Abstract

**Background and Objectives:**

Older people are likely to transition to a new home closer to family who can provide assistance or to long-term residential care as their health declines and their care needs increase. A minority choose to move to “age-friendly” housing before the onset of disability, but the majority prefer to “age in place” and defer moving until health crises compel a transition. Older people living with dementia are likely to move into residential care, but not much is known about the role they play in decision making around these moves. This qualitative study addresses this gap in knowledge by examining how a rare cohort of “older old” people, most with some level of cognitive impairment, were involved in decisions surrounding assistance seeking and moving to a care home.

**Research Design and Methods:**

Thematic analysis of qualitative interview data from Cambridge City over-75s Cohort (CC75C) study participants aged 95 years and older, who had moved in later life, and their proxy informants (*n* = 26).

**Results:**

Moves at such an old age were made due to a complexity of push and pull factors which had layered dynamics of decision making. In most cases (*n* = 22), decision making involved other people with varying degrees of decision ownership. Only four older people, who moved voluntarily, had full ownership of the decision to move. Many relatives reported being traumatized by events leading up to the move.

**Discussion and Implications:**

“Older old” people are sometimes unable to make their own decisions about moving due to the urgency of health crisis and cognitive decline. There is a need to support relatives to discuss moving and housing options at timely junctures before health crises intervene in an effort to optimize older people’s participation in decision making.

**Translational Significance:** Frail older people are likely to relocate to long-term residential care. Research on how the oldest members of our society navigate this transition is minimal. This article presents qualitative data from a rare cohort of the oldest old to illustrate the complex interacting push and pull factors triggering a move and documents the limited involvement of those living with dementia in decisions about moving.

The transition to a new home closer to family or to long-term residential care often becomes necessary for older people with increasing levels of frailty. Those who move closer to family pulled by their need for assistance are typically “light help seekers” with mild to moderate disability. Older people with multiple comorbidities and high dependency in instrumental activities of daily living (IADLs) are typically “heavy help seekers” and are more likely to be pushed by their increasing frailty to move into care (1).

A minority of older people voluntarily move closer to family for support in anticipation of the need for assistance (2,3) well ahead of a health or social care crisis when they have control over their decision to relocate (4,5). At this point, older people can be classified as making a “positive choice” (5). The vast majority of older people prefer to “age in place” for as long as possible, deferring moving until a health crisis compels it and their capacity for involvement in decision making is compromised (1,6).

Frail older people who have aged in place can make the decision to move into residential care voluntarily, typically in circumstances where they can view it as a “rational alternative,” justifying the move as an altruistic act that will protect their informal carers from the burden of their increased dependency (5). However, more often the decision is made “with others,” or even “by others,” with some evidence of older people’s views and preferences being overridden and moves being organized without consent (5,7–9). Relatives and health and social care professionals are usually involved in such decision making (5,10–12) and exert more power when the older person needs help with basic activities of daily living (BADLs) and IADLs (13–15). Informal carers’ goals in these circumstances prioritize safety and security, while the older person may well strive to maintain their autonomy (16). Ultimately, decision making around moving in later life involves compromise and conflict (17), moral persuasion (18), and judgments about potential gains and losses (1,7).

The likelihood of moving involuntarily increases with age and those aged 80 years or older are identified at high risk (3,19). Moves within this age bracket are driven by the push and pull of major life events (eg, serious injury from a fall, hospitalization, or death of spouse or informal carer) that threaten functional competence (3,20,21). When chronic disability or illness overwhelms the ability of family and others to provide sufficient care, “institutional pressure” arises for the older person to move into a residential or nursing home (22,23). In these situations, there is a high risk that the decision to move is a “fait accompli” or a “discredited option,” where the older person agrees to a move under the false assumption that it can be reversed, which is used to secure their compliance (5).

As they are often excluded from interview studies, less is known about how older people living with dementia are involved in the decision to move into residential care (20). This paper addresses this gap in the literature and aims to deepen our understanding of how “light” and “heavy” help seekers living with variable levels of cognitive disability are involved in decisions when making voluntary and involuntary “assistance seeking” and “residential care moves.” To achieve this objective, we analyze qualitative interview data collected in a representative UK-based longitudinal study of aging when the surviving cohort, then aged 95 years and older, were facing various push and pull pressures to relocate.

## Design and Methods

### Qualitative Interviews in a Longitudinal Cohort Study of Aging

At Year 21 (2006–2007), surviving participants of the Cambridge City over-75s Cohort study (http://www. cc75c.group.cam.ac.uk/) were invited to take part in an additional qualitative interview to explore the lived experience of aging that the main survey questionnaire was unable to record. The overarching aim of the qualitative study was to explore “what it is like to be so old.” A secondary aim was to learn more about transitions in care and relocation in very old age (topic-guide: http://www.cc75c.group.cam.ac.uk/documentation/additional-data-collection-formats/). Cambridge Research Ethics approval was obtained for the qualitative data collection.

### Ethical Approval

Each CC75C study phase was approved by Cambridge Research Ethics Committee (relevant reference numbers: 06_Q0108_87 and 08_H0308_3) and participants’ and proxy informants’ consent was re-sought at each new survey and for the informant interviews after participants had died.

### Purposive “Critical Case” Sample

Forty-two of 48 surviving participants took part in the qualitative interviews at Year 21. Using study archives to check address details before and after Year 21 data collection, we identified a subset of 26 “critical case” (24) participants, aged 95+, who moved. Seeking out groups or settings where the phenomena of interest is likely to have taken place is a recognized strategy for purposive sampling in qualitative research (25).

### Triangulation of Data by Person

**Figure 1.**
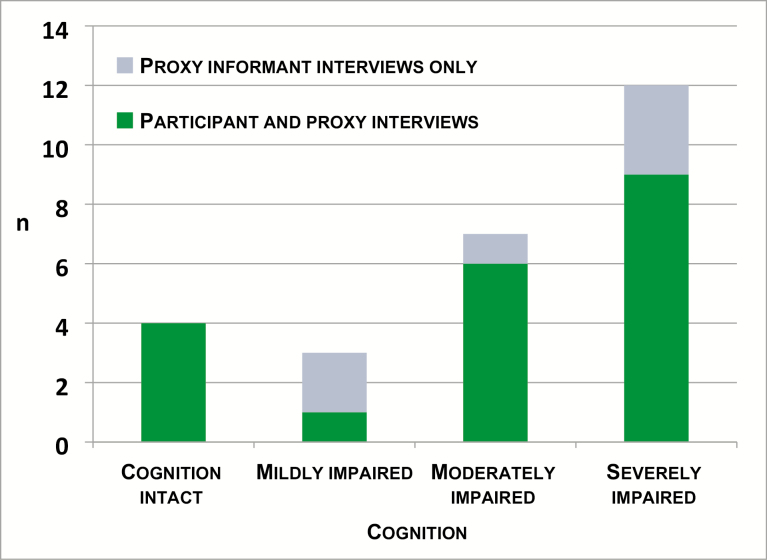
Qualitative interviews available for analysis by categories of cognitive function (*n* = 26 aged 95–100 years old) at year 21 (2006–2007).

Twenty of the 26 participants who relocated took part in qualitative interviews; six were too frail to speak for long periods. Twenty-two of the cohort participants who relocated had some level of cognitive impairment. In view of these communication difficulties, a key carer, normally a close relative, was interviewed as a proxy informant using an adapted topic guide which asked them what they thought their older relative would think or feel about the topic as well as their own perspective. A total of 29 proxy interviews were completed (two proxy interviews were completed for three or the 26 participants). Twenty-five proxies were interviewed individually and four were interviewed jointly with the cohort participant. A challenge when interviewing dyads and individuals with cogitative impairment is how to retain the older person’s voice so that their status as an “agent” is not obscured (26,27). To avoid this, the paper presents findings as emergent from either the older person’s narrative or the proxy informant’s. This approach increases clarity and ensures that older people’s accounts are not conflated with those of their proxy informants (27). A summary of the qualitative interview sources across the categories of cognitive function of participants in the cohort is presented in Figure 1.

### Interviews and Documents

Interviews were conducted in participants’ or proxies’ place of residence, audio-recorded, and professionally transcribed. Interviews lasted between 1 and 1½ hours. Additional data sources available for analysis include (1) transcribed extracts from audio-recordings of main survey interviews, (2) field notes written up after interviews, and (3) emails and handwritten letters of correspondence from proxy informants. The triangulation of data by person and source adds integrity to the analysis because issues could be revisited and clarified using the technique of “member checking” (28).

### Thematic Data Analysis

All data were anonymized and identifying characteristics removed. Where quotes are presented in the text below pseudonyms are used to maintain anonymity. One researcher (F.S.), trained in qualitative methods, read the anonymized transcripts and developed a first-order coding framework using a constant comparative method (29) to identify commonalities and non-confirming cases. Codes were developed inductively from the verbatim text; for example, the code “trauma” used to code proxy interviews occurred naturally in the spoken text of eight interview transcripts. Reliability checks were carried out by a second researcher (J.F.) and discussed with the full team at monthly project meetings along with the findings of a review of the related literature. Transcripts were imported into NVivo and charted into a framework matrix under six thematic headings: (1) housing decision, (2) reason for move, (3) housing transition, (4) attitudes to moving, (5) views on life and care choices, and (6) reflections on the experience of moving. Participants’ levels of cognitive and physical disability were recorded in the same thematic column as housing decision, along with any contextual data about how the decision was made from proxy interviews. Themes were compared against one another to check for saturation using a constant comparative method that mirrored the approach outlined by Constantinou and coworkers (30). The final analysis distinguished (1) circumstances of the move, (2) who had involvement in decision making, (3) the timing of the decision (ie, was it planned or made in response to a crisis), and (4) the older person’s reflections on and experience of moving. The analysis team worked independently, in parallel and together at various steps of the analytic process.

### Rigor

The authenticity, rigor, and quality in the collection and analysis of the CC75C qualitative data were achieved by (1) writing reflexive field notes after interview, (2) triangulating data collection by person, (3) “member checking” key events with proxy informants, and (4) application of the constant comparative method to ensure concept saturation (31). The development of an audit trail of conceptual and thematic thinking and methodological decisions by the lead data analyst (F.S.) also increased rigor.

### Socioeconomic Context

All of the participants and proxies interviewed were born in the United Kingdom and lived in Cambridge City, an urban district 50 miles northeast of London. The study samples were recruited from general practices purposively sampled to represent Cambridge’s social inequalities. Life expectancy at birth is higher in Cambridge than in England as a whole, but the difference is statistically significant only for women, who are likely to live 5 years longer than men (32). At the time of data collection, Cambridge had a relatively ethnically homogenous “white” population of approximately 119,000 persons comprising mainly non-manual and skilled manual social classes (32).

## Results

The samples were mainly women and most (*n* = 23) were widowed including the only man. The median age of the sample was 97.1 years (interquartile range 96.2–98.4) and the age range was 95–101. Twenty-two (84%) had some level of cognitive impairment and 12 were severely impaired. The majority (22/85%) needed help with BADLs, two (8%) needed help only with IADLs, and three needed no help with either. Most (*n* = 18/69%) had been employed in non-manual or skilled occupations (see Supplementary Table 1 for full details of the sample [*n* = 26] characteristics).

The cognitive status of participants is indicated in the text after quotes by the abbreviations “SCI” denoting severe cognitive impartment, “ModCI” moderate cognitive impairment, “MCI” mild cognitive impairment, and “NCI” no cognitive impairment.

Most proxy informants were women (22/29). Proxies were daughters (*n* = 14), sons (*n* = 6), other relatives (three children-in-law, two nieces, and one sister), a friend, and two care home managers. The survey did not collect demographic details for proxy informants. All were in regular contact with their relative, often visiting them more than once a week.

By the time they died, all of our sample had moved to a care home except for two: one of these died in a long-stay hospital ward before a nursing home place was found, and the other had moved to relatives and lived with them until almost the end of her life but died in hospital. Two others had moved to live with family members before they subsequently moved into a care home and two had initially moved to sheltered accommodation before their move into care. Three participants moved from one care home to second one before they died. All but two of the moves were at least in part prompted by health crises, often falls, leading to hospital admission, many compounded by worsening cognitive impairment. Given this context, it is not surprising that many of the proxy informants (11) described how traumatic it was to be supporting frail older relatives through any kind of move in later life:

Rose Baker’s daughter: {A week before moving} she fell from, she thinks now, from about half way up the stairs. And when I went round at sort of 9 o’clock in the morning I found her at the bottom of the stairs. […] It was, I mean, it was very traumatic. *(Woman, aged 98, IADLs, NCI, moved into sheltered housing later moves into care after hospital then to a second care home)*

Flora Chamberlain’s daughter: I have to [..] see if I can get a nurse to be with her while I go [home] because she’s always sort of saying to me “[..], “Don’t go without me.” […] Yes, her phrase when she actually first went in there [the second care home] was “You’ve done for me.” […] and it’s pretty well done for her in that now she is, you know they’ve been sedating her a bit. *(Woman, aged 97, IADL and BADLS, SCI, moved in with family then to a care home then moved to a second care home when the first one closed)*

**Figure 2.**
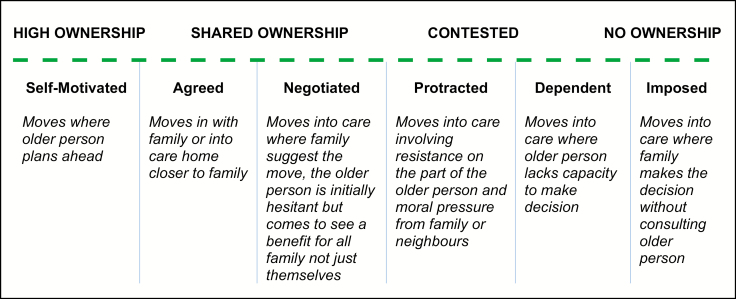
Taxonomy of the oldest olds involvement in decision making in voluntary and involuntary moves identified in the CC75C qualitative data.

Findings from the qualitative analysis are organized in two subsections to reflect the degree of voluntariness: voluntary (*n* = 7) and involuntary moves (*n* = 19). In these subsections, data are presented to illustrate the push–pull triggers for moving, the level of ownership participants had of the decision to move, and how decision-making patterns differed by level of need. Figure 2 presents a taxonomy of the six forms of decision making we identified in the activation of voluntary and involuntary moves.

### Voluntary Moves: Into Sheltered Housing, in With Family and Into Residential Care

#### High ownership decision making

Light help seekers made “self-motivated” decisions to downsize from homes that no longer met their needs:

Rose Baker: When I put my name down for here it wasn’t all that long… I should have moved years ago. I could have come here, I think, before.

Interviewer: You mean you were beginning to feel it was a bit difficult in your house?

Rose Baker: Well, …all the windows, three bedrooms, stairs… (*Woman, aged 98, IADLs disability, NCI, moved into sheltered housing, later moves into care after hospital, then to a second care home*)

However, triangulation of data showed that light help seekers’ “self-motivated” decisions would likely not go ahead if they violated carer safety concerns. For example, Rose Baker had a fall the day before she moved which meant that her daughter took the final responsibility for deciding if the move should go ahead:

Rose Baker’s daughter: We had to make a decision as to whether to carry on with the move or not. But the flat that she’s moved into is all on the same level and if she’d stayed where she was, you know, the bathroom’s upstairs, the kitchen’s downstairs and the bedroom’s upstairs, so we made the decision in the end to carry on and move her. *(Woman, aged 98, IADLs disability, NCI, moved into sheltered housing, later moved into care after hospital, then to a second care home)*

Those with increasing frailty also made “self-motivated” decisions to move in with family, but only did so when they were emotionally overwhelmed by the efforts of trying to maintain their autonomy, or by loneliness:

Charlotte Smith’s daughter: Mum was [..] very ill […] we brought you [addressing her mother] back here and you [addressing her mother] couldn’t get out of bed […]. And then [..] you [addressing her mother] wanted to go back [home]. And then one day we popped in on you and you were [..] sitting on the back doorstep, very sad. And you said: “I want to come and live with you.” *(Woman, aged 98, IADL’s only MoDCI, moved from own home in with family, and later moves into a care home)*

Stella Thatcher: Oh, I was very grateful that they [family] would take me here because it was very lonely. *(Woman, aged 96, BADLS and IADLs, SCI, moved from own home in with family. No further moves)*

Family members expressed relief when their relative took ownership of the decision to move, accepting they needed more help and could no longer manage alone:

Daughter of Stella Thatcher: I was very pleased that she was the one that made the decision to... (move in with us). Yes. I mean she..., I think I told you last time, she just walked out with a carrier bag with her nightie in and said she was coming here. *(Woman, aged 96, BADLs and IADLs, SIC, moved from own home in with family. No further moves)*

#### Shared ownership decision making

One heavy help seeker “agreed with family” proposals for a move to residential care closer to her daughter after she had lost local support networks, but she later regretted making this decision:

Florence Potter: Well, (moving to this care home) it was a bit awkward because (my daughter) had phoned and said [..] to come to Cambridge would take about three hours, whereas [..] “If you’re… [..] near me I can get to you in about twenty minutes” [..] She doesn’t often come. ’Cos it was a damn silly thing I did to come here from Cambridge. [..] And so, I mean, what with losing these friends. [Pause] Yeah. I thought “Well, I might as well go and… yeah, make a do of it altogether.” *(Woman, aged 98, BADLS + IADLs, SCI, moved from own home to care home closer to family, and later moves to a second care home)*

Moves among heavy help seekers who experienced falls, hospitalization, and cognitive decline sufficient to motivate “carer perceived risk” were “negotiated by family” who encouraged participants to view moving as a “rational alternative”:

Patricia Miller’s daughter: I went in one afternoon and she didn’t really know me, and she was in quite a state, […]… then I didn’t feel that I could rely on her to do things, like I’d leave her....[…], you know, everything was there, everything was labelled, notes were left, but they weren’t being followed. *(Woman, aged 100, BADLs and IADLs, SCI, moved from sheltered housing to residential care following a fall)*

Patricia Miller: Well, when I first thought about it, I wasn’t particularly keen. Then I thought about it and realised, for my family’s sake, to give them peace of mind. It would be the wisest thing to do ’cos they’d know that there was somebody on hand, if I needed it. And that’s the way to look at it. Because it would be less worry for them. *(Woman, aged 100, BADLs and IADLs, SIC, moved from sheltered housing to care home near family following a fall)*

Some “negotiations by family” met with resistance had to be revisited at fraught hospital bedsides and were subject to scrutiny from social workers:

Daughter of Prudence Sawyer: It was our big problem (getting mother to agree to move into a care home near us). And then we were talking about it (in the hospital) and said “Well, what if you move down to Seaside Ville? We’ve found you somewhere in Seaside Ville. It won’t be a flat.” We had to make that definitely clear to her. “But would you consider it?” And she said “Yes.” And the social worker said “Well, I’m gonna have to see your Mum to see that’s definitely what she wants.” So we went back into the ward and asked her the same question and she said “Yes”.[…] And that was it. *(Woman, aged 97, BADLs and IADLs, SIC, moved from own home to residential care closer to family following hospitalization)*

Gaining agreement for a move into care was eased where the older person could feel a connection to the place they were moving in to, either by having had a period of respite care there or knowing a person who worked at the care home:

Primrose Turner’s daughter: Very difficult (gaining agreement from mother about her move into care). And I think if my son hadn’t worked at the home, he works at that home [as a chef], that perhaps we still wouldn’t have got her there. After she’d been there a couple of weeks, I suppose, I said to her “I don’t think you can manage in your flat now” and she said “No, perhaps I can’t, I don’t think I can.” And she said “It’s nice having the company.” *(Woman, aged 100, BADLs and IADLs, SIC, moved from own home to residential care after repeated falls)*

Patricia Miller’s daughter: She settled in quite well. Yes. Oh yes, no problems at all. At least she knew where she was going, and she knew a lot of the people, the staff. That’s where she did her respite. *(Woman, aged 100, BADLs and IADLs, SCI, moved from own home to residential care)*

### Involuntary Moves Into Residential Care

Heavy help seekers with high levels of disability and cognitive impairment made involuntary moves directly into residential care following an injurious fall, a period of hospitalization, confusion, or incontinence:

Mary Taylor’s daughter: She had a fall after Christmas, she was out and literally the wind blew her over. She broke her wrist. So of course, she was hospitalised [….] She was also getting extremely confused. Getting a bit incontinent and all sorts of things were happening. *(Woman, aged 95, BADLs and IADLs, SCI, moved from own home to residential care after fall and hospital admission)*

Margaret Butcher’s daughter: Mum went into hospital following another more serious fall and subsequently into respite care and then into full-time residential care. *(Woman, aged 97, BADLs and IADLs, SCI, moved from own home to residential care as dementia worsened)*

Millicent Lorrimer’s sister: I think it was Christmas Eve, she was taken ill. […] she fell out of bed, and of course then I couldn’t move her [..] Anyway [..] I dialled for the ambulance and [...] well, they said that she must go to hospital and that was that. *(Woman, aged 95, BADLs and IADLs, SCI, moved from own home to residential care after fall and hospital admission)*

#### Protracted decision making

Decision making for five participants was ‘protracted’ which was characterized by (1) prolonged resistance to moving on the part of the older person, (2) moral pressure from neighbors, and (3) family invoking the powers of health professionals. In the following example, neighbors called family members directly and alerted a local older people’s charity helpline that the participant was found in the street in her nightclothes; her family finally requested a formal referral from an old age psychiatrist:

The (care home) has been a bone of contention, she (mother) has resisted most forcefully [..] There have been some disturbing behavioural incidents this past week or so. Several of Mum’s neighbors have, as you know, been contacting Age Concern etc., [..], but recently my sister and I are having telephone calls direct. [..] Slightly critical in nature [..] They all say she needs residential care [and] is a liability to those around her [..] I am working towards a permanent placement in residential care. In case Mum is still resistant I would appreciate you *(addressing her mother’s social worker*) arranging for the psychiatric geriatric consultant to visit Mum while she is in respite care. It is a shame that it has come to this, but Mum has been in a very confused state which has become much worse recently. *(Extract from a letter to social services shared with the research team by Margaret Butcher’s daughter) (Woman, aged 97, BADLs and IADLs, SIC, moved into residential care as dementia worsened)*

#### Dependent decision making

*Dependent* decision making was observed in two cases and one of these participants (who moved into residential care following hospitalization for shock due to the sudden death of her husband) was aware of her vulnerability in the decision-making process:

Agatha Cooper: Yeah. ……They wouldn’t let me go [home] to have a [shower]. Just straight out of hospital here.

Interviewer: Had you wanted to go back to your bungalow then? Or not really after your husband wasn’t there?

Agatha Cooper: I don’t know. I don’t know. I can tell you that I was in such a daze. (*Woman, aged 98, BADLs and IADLs, ModCI, moved from own home into residential care after husband died*)

#### Imposed decision making

*Imposed* decision making was observed in four cases, with one of these participants having a strong awareness of having their preferences overridden:

Beatrice Skinner: They wouldn’t let me come back here, no, they wouldn’t let me go back to my bungalow, I mean. Because they were saying “Oh, you should go, Mum”, […] Perhaps they were right. But there’s no getting out of it when you’ve made that decision. *(Woman, aged 97, BADLs and IADLs, MCI, moved into nursing home due to ill health)*

Some family members who *imposed* the decision to move onto their relatives were candid about their inability to cope with the impact of rapid cognitive decline and fecal incontinence which was a clear trigger for an *imposed decision*:

Loretta Fowler’s son: She was going downhill rapidly even at that stage. And one day she came round here and she messed herself. So that was a nice little “how do you do” and after that I thought to myself “Well, I can’t contend with this.” I cleaned her up as best I could, but I couldn’t really contend with this and that was when we decided she had to go into a home. *(Woman, aged 97, BADLs and IADLs, SIC, moved from own home into residential care after husband died and dementia had worsened)*

## Discussion and Implications

Voluntary moves were observed among “light to moderate help” seekers (IADLs disability only and ModCI), seeking to downsize to smaller more manageable homes. Some “heavy help” seekers (BADLS and IADLs disability and SCI) also made voluntary moves in with family or into residential care homes closer to family. In this transition pathway, decision making ranged from “self-motivated,” to “agreed with family” or “negotiated by family.” In the latter form, the older person is initially hesitant about moving but decides a move will be of benefit for themselves and the family, which is broadly consistent with the concept of a “rational alternative” (5).

Involuntary moves were observed among “heavy help seekers” (with BADLS and IADLs disability and SCI) some of whom actively resisted moving into residential care or had diminished capacity for engagement in decision making. This group correspond with Golant’s (1) delineation of “heavy help seekers” insofar as they experienced multiple transitions (33) before a final move into care permanently. In this pathway, the decision to move was either “imposed by family” (unwilling to deal with incontinence, risks of further falls, burden of care), “dependant on family” (where older person lacked capacity), or “protracted/contested” as informal carer goals (for safety/security) clashed with the older person’s goals (maintenance of autonomy). In the latter context, family invoked the authority of old age psychiatrists and social workers to advance the case for relocation.

Moves were typically triggered by a crisis (injurious fall, incontinence, declining mobility, cognitive decline, loss of care) and often followed a period in hospital. This finding in our small sample is consistent with larger cohort studies showing that half of all new care home residents in the United Kingdom move in straight from hospital (21) and the findings of a recent systematic review on timing of moves among those living with dementia (20). Incontinence and advancing cognitive impairment also prompted a transition to a care home in our sample, in line with previous research (9).

Our findings complement and expand upon a previous tripartite taxonomy of ownership of the decision to move as either “by self,” “with others,” or “by others” (7) and the earlier work by Nolan and coworkers (5) who distinguish between “positive choice,” “rational alternative,” “discredited option,” and “fait accompli.” Within the CC75C rare cohort of people aged 95–101 years, most decisions about moving were made “with others” or “by others.” Informal carers who had most responsibility for CC75C participants played a dominant role in decision making, consistent with previous research examining the relationship between care giving and accruement of power (13–15,34,35). This dynamic sometimes put older people at risk of making decisions, or being the subject of others’ decisions about moving, that they later regretted. Our findings also add descriptive detail to the taxonomy of older movers as “light help seekers” and “heavy help seekers” (1). We elaborate these taxonomies by describing six processes of decision making that attempt to illustrate the nuances and complexity of decision making about moving in later life. Our taxonomy introduced the concept of “protracted” decision making where the goals of informal carers or safety or security came into conflict with the goal of autonomy for the care recipient, which is also highlighted in the care giving literature (16). “Protracted” decision making involved “moral pressure” from external agents, and health professionals: a finding that is consistent with studies of downsizing where older people’s decision to move “is oftentimes motivated and propelled by moral persuasion” from family and the wider community (18).

The likelihood that older adults are not part of the decision-making process for transitions into care has been highlighted previously (5,10,12,36). Our analysis shows that where the older person was aware of decisions being imposed they felt resentment, confirming earlier work by Nolan and coworkers (5). But for others in our sample the loss of control was accepted philosophically as part of the process of aging. These differences may be associated with how the transition is managed, as well as personal reserves of resilience, and should be the subject of further research.

Our data show that older people living alone make self-motivated decisions to move to where there is more help which is consistent with previous qualitative research (5,7). The main barrier to moving that we observed was a mind-set of independence and attachment to place; the main facilitator was having social connections in the new place of residence which has been highlighted in the theoretical literature as significant (1). Most importantly, our data suggest that the “fait accompli,” or what we term “imposed decision,” has not, as Nolan and coworkers (5) had hoped, become a “thing of the past” (p. 273).

### Limitations and Future Research

Our retrospective analyses is limited by the fact that the data were not triangulated by method: using observational methods to follow older people through their move experience would have allowed us to determine what effect making an unplanned move into residential care had on their well-being and explore the extent to which they could achieve “residential normalcy” (1). Societal awareness of dementia and the residential care landscape has changed since the CC75C data were collected and future research should aim to determine the extent to which older people, particularly those living with dementia, are supported to be involved in decision making around moving into care. More research is needed to gather the views and experience of all stakeholders involved in the decision-making process of older peoples’ moves as this has not been well documented to date and would provide the basis for developing supportive interventions for both relatives and older people.

## Supplementary Material

Supplementary data are available at *Innovation in Aging* online.

Supplementary Table 1. Characteristics of *n* = 26 CC75C study participants with qualitative data about moving.

igz030_suppl_Supplementary_Material

## Funding

We thank all the past CC75C sponsors for financial support spanning the decades since the Medical Research Council enabled the first follow-up survey (see website for full list of grants: http://www.cc75c.group.cam.ac.uk/background/grants/). A BUPA Foundation grant supported data collection and Abbeyfield Research Foundation funded analysis for this paper. A Welcome Trust Small Arts Fund for Engaging Science funded CC75C’s collaboration in a theatre production, about moving into a care home, which enabled wider public engagement with the research topic. CC75C was a member study of the National Institute for Health Research-funded Collaboration for Leadership in Applied Health Research & Care (CLAHRC) for Cambridgeshire and Peterborough. No funder played any role in the study design; collection, analysis, and interpretation of data; or in writing the report or the decision to submit the article for publication. All researchers were independent from funders.
