# Supplementary material for: When Frail Older People Relocate in Very Old Age, Who Makes the Decision?
Source: Innov Aging. 2019 Sep 6;3(4):igz030. doi: 10.1093/geroni/igz030 (PMC7127322; doi:10.1093/geroni/igz030)
Supplement: igz030_suppl_Supplementary_Material [file igz030_suppl_supplementary_material.docx]

Supplementary Table 1. Characteristics of n=26 CC75C study participants with qualitative data about moving

|  |  | (n=26) | |
| --- | --- | --- | --- |
| Age (years) |  |  |  |
| Mean (SD) |  | 97.4 (1.5) | |
| Median (IQR) |  | 97.1 (96.2 - 98.4) | |
| Range |  | 95.4 - 101.4 | |
| Age when 1^st^ moved |  |  |  |
| Mean (SD) |  | 95.7 (2.9) | |
| Median (IQR) |  | 95.9 (94.0 - 98.1) | |
| Range |  | 89.8 - 99.8 | |
|  |  | n | (%) |
| Sex |  |  |  |
| Male |  | 1 | (4) |
| Female |  | 25 | (96) |
| Marital Status |  |  |  |
| Married |  | 0 | (0) |
| Widowed |  | 23 | (88) |
| Separated/Divorced |  | 1 | (4) |
| Single |  | 2 | (8) |
| Education  (school leaving age) |  |  |  |
| <15 years of age |  | 9 | (35) |
| ≥15 years of age |  | 17 | (65) |
| Social Class ^*^ (occupation) |  |  |  |
| Non-Manual |  | 18 | (69) |
| Manual |  | 8 | (31) |
| Cognitive function ^†^ |  |  |  |
| Normal cognition |  | 4 | (15) |
| Mildly impaired |  | 3 | (12) |
| Moderately impaired |  | 7 | (27) |
| Severely impaired |  | 12 | (46) |
| Disability in ADLs ^‡^ |  |  |  |
| No disability |  | 2 | (8) |
| IADL disability only |  | 2 | (8) |
| IADL + BADL disability |  | 22 | (85) |

***Notes for Table 1****:*

Column percentages may not total 100% due to rounding each percentage.

* Social class categorised following contemporary UK Office of National Statistics grading of occupation reported at baseline interview: Non-Manual = I, II or IIIa, Manual = IIIb, IV or V.

**†** Mini-Mental State Examination complete scores, plus score category imputation and dementia status if incomplete, categorised 0-17 severe cognitive impairment, 18-21 moderate cognitive impairment, 22-25 mild cognitive impairment, 26-30 normal cognition.

**‡** IADL = Instrumental Activities of Daily Living; BADL = Basic (personal) Activities of Daily Living
